# Internet Addiction and its Predictors in Guilan Medical Sciences Students, 2012

**DOI:** 10.5812/nms.11626

**Published:** 2013-06-27

**Authors:** Shahla Asiri, Fatemeh Fallahi, Atefeh Ghanbari, Ehsan Kazemnejad-leili

**Affiliations:** 1 Department of Community Health Nursing, Guilan University of Medical Sciences, Rasht, IR Iran; 2 Social Determinants of Health Research Center, Guilan University of Medical Sciences, Rasht, IR Iran; 3 Department of Health Statistics, Guilan University of Medical Sciences, Rasht, IR Iran

**Keywords:** Internet, Internet addiction, Students, Iran

## Abstract

**Background:**

Internet is one of the technologies of the modern era that is being extensively used around the world. It is believed that excessive Internet use can be pathological and addictive. Though, academic use of the Internet is primarily intended for learning and research, students are one of the groups at risk of Internet addiction.

**Objectives:**

Due to the expanding use of Internet among the university students, this study was conducted to examine the Internet addiction and its predictors among Guilan University of Medical Sciences students.

**Materials and Methods:**

A cross-sectional study was conducted on 583 students during the first semester of 2012. A two-stage stratified random sampling was conducted and a two-part instrument was used for data collection. The first part of the instrument was consisted of questions about demographic characteristics and the second part was the Young's Internet addiction inventory. Chi-square, Kruskal-Wallis testes, Spearman correlation coefficient and ranked logistic regression were used for data analysis.

**Results:**

About 5.7% of the students were moderately dependent to the Internet, while 44.1% were at risk for Internet addiction. Significant relationships were observed between the Internet addiction with age (P < 0.001), gender (P < 0.001), marital status (P < 0.001), major (P = 0.016), Grade point average (P = 0.017), semester of studying (P = 0.009) and student residence place (P = 0.014). However, no significant relationship was observed between the internet addiction score and level of discipline, parental job status and education level or the students’ accommodation.

**Conclusion:**

About half of the participants in this study were at risk of Internet addiction. This finding can be a warning sign for the authorities in universities to pay more attention to this issue. A wide range of education along with empowering programs may be needed to inform the university students about the advantages and disadvantages of internet and the correct manner of using it.

## 1. Background

Internet is one of the technologies of the modern era. Unique features such as easy access, low cost, being affordable and anonymity in cyberspace have made it extremely welcomed around the world (1). Internet access in Iran has been growing according to Internet World Stats (IWS): Number of Iranian Internet users is 42,000,000 , equivalent to 53.3% of Iran's population, are Internet users and this means Iran is in the third place among 20 countries with many internet users. But in terms of Internet Impact Factor is located in the tenth grade , and according to the International Telecommunication Union is Classified as the world average countries (2).

However, some academics believe that excessive Internet use can be pathological and addictive and can be classified under the generic label of technological addiction that is a subset of behavioral addictions (3).

This issue has been discussed under different titles such as behavioral dependence to Internet, Internet abuse, problematic Internet use, pathological Internet usage, and cyber and virtual addiction, however, ‘Internet addiction’ (IA) is the most common term used in literature (4). According to Chou et al. Internet addiction can be defined as using Internet in a way which results in psychological, social, academic and professional problems (5).

Though, academic use of the Internet is primarily intended for learning and research, students are one of the groups at risk of Internet addiction. University students are facing with a new life that may be challenging to be compromised with and finally result in feeling of loneliness, anxiety and depression. In addition, lack of parental controls, access to Internet and inevitable need of using it, having enough time as a result of a flexible curriculum and lack of control over what they are doing on the Internet make them more vulnerable to Internet addiction (6).

Frangos quotes from Young (1996) that the prevalence of Internet addiction in the world-wide general population is five to ten percent (7).

Studies show that Internet addiction is a global phenomenon with different prevalence that ranges from 5 to 25 percent among the university students in United States, China, Korea, United Kingdom, Australia, Taiwan, Japan, South Korea, and Europe (8).

Studies show that demographic factors, some specific personality traits, having a history of psychological disorder such as anxiety, obsessions, addictions, depression and social phobia, and the type of Internet use, are predictors of Internet addiction (9). A recent study on Internet addiction mainly emphasizes on three types of factors including: personal, socio-psychological and Internet related factors (10).

It has been reported that Internet addiction is associated with social phobia, attention deficit disorder, hyperactivity, bipolar disorder, problems in interpersonal relationships, anxiety and impulse control disorders, suicide, aggression, depression and high levels of excitement or euphoric reactions (11). On the other hand, some studies have examined the relationship between demographic variables and Internet addiction and indicated that gender, age, unemployment, parental education level and marital status are significant predictors of IA (12). However, results of studies on individual and social factors predicting Internet addiction are sometimes contradictory. In some studies, the age between 19 to 30 years (13) and in other studies adolescence (14, 15) has been identified as a risk factor for IA (11).

It has also been reported that Internet addiction is significantly associated with male gender (4, 7, 15). However, there are also conflicting studies (14). Some studies also indicate that financial issues and familial and psychosocial conflicts are predisposing factors for IA especially for those students who are struggling with stressful developmental demanding conditions (6). Recently several Iranian researchers have investigated the influences of family on the use of internet (7), relationship between internet usage and social isolation among Iranian students (14), internet addiction in local general populations (14, 16) and Internet addiction in medical students (16, 17). One study has also conducted on psychometric properties of Young internet addiction test (18). However, no study compared the situation of IA between the students with different fields of study especially the nursing and midwifery students.

## 2. Objectives

Due to the role of cultural factors in IA (5), expanding uncontrolled use of internet in community and among the university students and the need of clarifying the predictive factors of IA, this study was conducted to examine the IA and its predictors among Guilan University of Medical Sciences students.

## 3. Materials and Methods

A cross-sectional study was conducted in the first semester of 2012 in Guilan University of Medical Sciences, Guilan, Iran. Sample size was estimated based on a previous study that reported the prevalence of IA to be 10.8% (14).Then 592 samples were estimated to be needed based on the following parameters (α = 0.05, β = 0.95 and sampling error of 2.5%) and 652 samples were selected from a total of 3422 students (2336 males and 1086 females). However, 583 samples were entered in the final analysis ( 69 questionnaires were responded incompletely and were excluded). A two-stage stratified random sampling was conducted. At first, the number of samples needed of each discipline and each grade was calculated and then the needed samples were selected randomly using the list of student in each discipline and each grade.

Being at least in the second semester of studying and consent to enter the study were selected as inclusion criteria. A two-part instrument was used for data collection. The first part was consisted of 10 questions about demographic characteristics including the students' age, gender, academic major, semester of studying, level of discipline, accommodation, place of residencey and marital status and parental job and education levels. The Persian version of Young's Internet addiction questionnaire was used as the second part of the instrument.

The Young's IA questionnaire consists of 20 items ranked on a 6 options Likert scale from never = 0 to always = 5, with the minimum and maximum score from zero to 100, respectively. The obtained scores were categorized into four categories of healthy (score 0-19), at risk (score 20-49), moderate dependence (score 50-79) and severe dependence (score 80-100) (18).

Reliability of Young's IA questionnaire was previously confirmed using half split method (r = 0.72) and calculating the Cronbach’s alpha that was 0.71 (18). Psychometric properties of the Persian version of this questionnaire was done by Alavi et al (17). They confirmed the instrument’s convergency and content validity, and calculated its reliability using test-retest method (r = 0.82), half split method (r = 0.72) and internal consistency (α = 0.88). The best cut-off point of the instrument was the score of 46 (17).

Data analysis was done using SPSS version 16 (Statistical Package for Social Science). As kolmogro-smirno test showed that the distribution of the data was not normal, nonparametric testes were used. Chi-square and Kruskal-Wallis tests were used for analysis of the status of IA in terms of demographic variables. Spearman correlation coefficient was used to calculate the correlation between IA and age. Also, ranked logistic regression was used to determine the predictive variables for IA. A P value less than 0.05 was selected as significant level in all the tests.

The study was approved by the Ethics Committee of Guilan University of Medical Sciences and obtained the necessary permissions from the research council, Guilan University of Medical Sciences. Aims of the study were explained to the subjects and they were assured of the confidentiality of personal information before starting the study and all signed a written informed consent. The researchers observed all ethical issues in accordance with the Helsinki Convention.

## 4. Results

A total of 583 students participated in the study of which 382 (65.5%) were female and 201 (34.5%) were male (Table 1). The mean age of students was 22.41 ± 3.64 with a range of 18 - 42 years. Most of the students were single (79.8%). Also the majority of the students were studying medicine (45.6%) and (26.8%) were nursing or midwifery students. Most of participants were undergraduate students (45.6%) and also mostly were in their 5th to 13th semester of studying. The mean of students' GPA (grade point average) was 15.89 ± 1.09. Most of the students (46.8%) were living at dormitory (Table 1).

**Table 1.** The Distribution of Some of Socio-Demographic Variables

Demographic variables	
Gender	Number (%)
Male	201 (34.5)
Female	382 (65.5)
**Marital status**	
Single	465 (79.8)
Married	118 (20.2)
**Educational level of mother**	
Illiterate & primary	64 (11)
Diploma & under diploma	320 (54.9)
Academic	199 (34.1)
**Educational level of father**	
Illiterate & primary	16 (2.7)
Diploma & under diploma	288 (49.4)
Academic	279 (47.9)
**Student accommodation**	
City	578 (98.8)
Village	7 (1.2)
Age, (Mean ± SD )	22.41 ± 3.64
**Semester ** **of studying, (Mean ± SD )**	5.22 ± 2.45
**Grade point average, (Mean ± SD )**	15.89 ± 1.09

**Figure 1.**
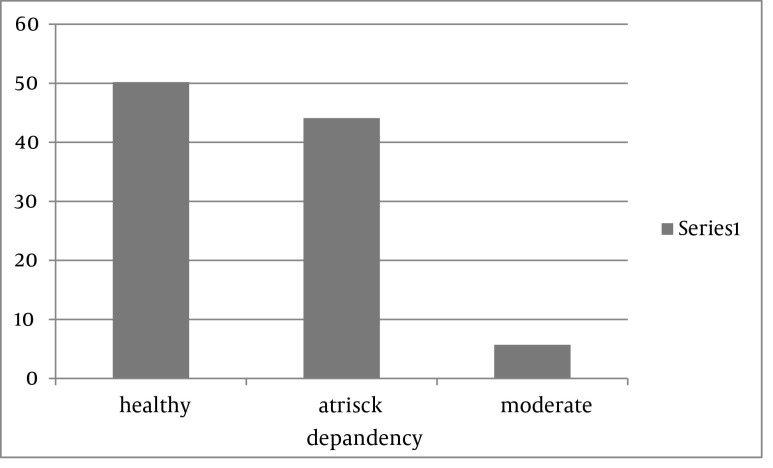
Distribution of the status of Internet addiction in students.

The mean score of IA was 22.13 ± 13.59 and the students were mostly (50.2%) healthy in term of the IA, however, 44.1% were at risk for IA (Figure 1).

Moderate dependence to Internet was higher in boys (10%) compared to girls (4.3%) and statistically significant relationship was detected between gender and dependence to the Internet (P < 0.001). The risk of addiction and moderate dependence were higher in singles than the others. As this rate was 48.4% and 6.5%, in singles and 27.1% and 2.5% in married students respectively (P < 0.001). Lower level of semester is associated with IA (P = 0.009). Grade point average (GPA) was 16.1 ± 1.04 in the healthy group, 15.82 ± 1.13 in at risk group and 15.44 ± 1.00 in moderate dependence group. This difference was statistically significant (P = 0.017) (Table 2).

**Table 2.** The Distribution of Internet Addiction in the Students in Terms of Demographic Variables

Variables	Healthy, No. (%)	At risk, No. (%)	Moderate dependence, No. (%)	Test result
**Gender**				< 0.001^a^
Male	84 (41.8)	97 (48.3)	20 (10)	
Female	209 (54.7)	160 (41.9)	13 (3.4)	
**Marital status**				< 0.001^a^
Single	210 (45.2)	225 (48.4)	30 (6.5)	
Married	83 (70.3)	32 (27.1)	3 (2.5)	
**Semester of studying**				0.009^a^
1-4	158 (27.1)	107 (18.4)	19 (3.3)	
5-13	135 (23.2)	150 (25.7)	14 (2.4)	
**Grade point average, (Mean ± SD)**	16.01 ± 1.04	15.82 ± 1.13	15.44 ± 1.0	0.017^b^

^a^ results of Chi-square test

^b^ results of Kruskal-Wallis test

Table 3 shows the distribution of IA scores of students in terms of demographic variables. The mean IA score in health and nursing majors was lower than in others. This difference was statistically significant (P = 0.016). There was a significant reverse relation between IA score and age (r = -0.014, P < 0.001). The mean IA score was lower in those who lived at dorm in comparison with the students who lived alone or with family. This difference was statistically significant (P = 0.014).

**Table 3.** Distribution of Internet Addiction Scores in the Students in Terms of Demographic Variables.

	Demographic variables, No. (%)	Internet addiction, (Mean ± SD)	P Value
**Level of discipline**			0.91^I^
Undergraduate	300 (51.4)	22.25 ± 12.71	
Master of science	17 (2.9)	20.35 ± 11.21	
Doctoral	266 (45.7)	30.25 ± 14.89	
**Academic major**			0.016^I^
Medicine^a^	266 (45.6)	22.11 ± 14.19	
Nursing^b^	156 (26.8)	21.28 ± 13.35	
Health^c^	45 (7.7)	18.41 ± 11.29	
Paramedical^d^	115 (19.7)	24.83 ± 13.04	
**Students’ father’s job**			0.705^I^
Official	143 (24.5)	22.66 ± 14.22	
Self employed	145 (24.9)	22.62 ± 14.58	
Farmer	35 (6)	24.62 ± 15.26	
Retired	260 (44.6)	21.23 ± 12.40	
**Student residence place**			0.014
Dormitory	273 (46.8)	13.03 ± 23.30	
Alone	101 (17.3)	15.63 ± 23.14	
With family	209 (35.8)	13.09 ± 2.11	
**Age**	r = -0.14^Y^		< 0.001

^I^ Kruskal-Wallis test

^Y^ Correlation coefficient

^a^ including medicine and dentistry students

^b^ Including nursing, midwifery and medical emergencies students

^c^ including public health, occupational health, environmental health and health education students

^d^ including students of operating room, anesthesia and radiology techniques

Regression analysis showed that among different variables, age of the students as a predictive

variable has an inverse correlation with IA in medical sciences students (Table 4).

**Table 4.** Regression Analysis of the Demographic Variables Associated with Internet Addiction

Variables	P value	CI 95% OR	OR	Standard error	Regression coefficient
**Age**	0.004	0.87 , 0.97	0.92	0.029	-0.085

## 5. Discussion

This study showed that only 5.7% of the students were moderately dependent on the Internet, while more than 44% were at-risk for IA. Globally, an average ratio of 2-5 millions of internet addicts has been estimated per 50 million regular users. In other words, about 5 to 10 percent of Internet users have IA (17). In a study on Turkish college students, 9.7% of them were Internet addicted (19). The same result has reported in an Iranian study (14). Also another study in Iran has reported that 10.8% of medical students were internet addicted,among them 2.8% and 8% had severe and moderate IA respectively (16). It seems that the statistics in different studies are nearly the same and slight differences may be attributed to differences in sample size and instruments.

The present study showed that age of the student is a predictor of IA. An indirect correlation was also observed between age and the score of IA. It means that students with lower ages are more vulnerable to IA. Although lee and Stapinski could not find a significant correlation between age and IA (20), ages under 20 years have reported to be a risk factor for IA in another study (16). A study by Fu et al has also showed that younger people used internet three times more than people with higher ages (9). Perhaps, some personality and age related features such as enjoyment and adventure made them more susceptible to IA. However, the aim and type of using the Internet and the contents available to the student also influence this issue (21).

This study showed that the rate of IA is significantly more in male than female students. Though in a few studies this rate was more in female students (5), the results of the current study is consistent with most of the previous studies that suggest the male gender as a predictor of IA (4, 7, 15), in a way that in one study the risk of IA for male students was 3.5 times more than female ones (16). In a review on IA, Chou et al. concluded that male internet users are more at risk of IA due to a stereotype using of sexual contents, however, female users may be asymptomatic or may present limited symptoms (5). It also seems that male students are more likely to become Internet dependent as they are more experienced in using the Internet, receive less parental supervision and use the Internet for entertainment purposes more than female ones.

The results of this study showed significant association between marital status and addiction to the Internet. Also the risk of Internet addiction was higher in single students. Being single, impaired family relationships and being divorced are well known risk factors for IA (5). Based on the Davis cognitive behavioral model, these people receive positive internal rewards such as a sense of competence and socialization while they are online that consequently intensifies the amount of using Internet (7).

At the current study, the mean score of IA was lower in the health and nursing students than other groups. Also students with fewer semesters of studying were more at risk for IA. One study did not find a significant relationship between field of study and IA but lower education level was associated with an increased risk for Internet dependence (16). Other studies have reported that IA is more common among freshman students (21) and students of computer science and engineering (1). It has also been shown that freshman students usually immerse in the internet during the first weeks but they gradually reduced their consumption after being fully familiar with the new environment (22).

The results showed a significant relationship between IA and semesters of studying and student’s GPA. In this study, average GPA of at risk and internet dependent students was less than the healthy group. This finding is consistent with Frangos study (7) and was in contrast with a study reported by Yen et al. (11). It seems that excessive internet usage may negatively affect the students’ academic achievement. Therefore, there should be some limitations in using the internet by the students especially for freshman ones.

At the current study, the mean score of IA was lower in those students who live in dormitory compared to others. The authors believe that maybe these students have less opportunities and facilities to connect to internet. Ghamari et al didn’t find association between IA and residence place (16).

Based on the findings, no relationship was found between the students’ level of internet dependency and factors such as students’ level of discipline, students’ accommodation, parental job status and parental educational level. In a study on predictors of heavy Internet use among Hong Kong university students, Kim et al. found that parents' education level was directly related with the amount of using Internet by the students but it was inversely related to the rate of internet abuse (1). Frangos et al. confirmed an inverse association between IA and the students’ financial and social class (7). This finding was in consistent with the reported of Vizeshfar who investigated the internet addiction among Larian net users (23). A previous study has also reported that studying in higher levels increases the students’ Internet skills and its duration of using (7).

Approximately half of the participants in this study were at risk of IA. This finding can be a warning sign for the authorities in universities to pay more attention to this issue. A wide range of education along with empowering programs may be needed to inform the university students about the advantages and disadvantages of internet and the correct manner of using it. Because age was one of the most important predictors of IA, the freshman student can be selected as the most important target population for these programs.

There are a variety of definitions and instruments to measure IA. Then, using a different instrument may result in different findings. Therefore further studies with different instruments are required to fully understand the extent of the problem and its predictive factors countrywide.

Also the main limitation of this study was relying to the students self-reports. Therefore an observational and longitudinal study is suggested to investigate the real situation of internet usage by the university students and its trend during their academic years.

## References

[1] Kim JH, Lau CH, Cheuk KK, Kan P, Hui HL, Griffiths SM (2010). Brief report: Predictors of heavy Internet use and associations with health-promoting and health risk behaviors among Hong Kong university students.. J Adolesc..

[2] (2012;). Usage and Population Statistics.. Middle East Internet Stats, Iran Internet usage, broadband and telecommunications reports.;.

[3] Widyanto L, Griffiths M (2006). Internet addiction: a critical review.. Int J Mental Health Addict..

[4] Byun S, Ruffini C, Mills JE, Douglas AC, Niang M, Stepchenkova S (2009). Internet addiction: metasynthesis of 1996-2006 quantitative research.. Cyberpsychol Behav..

[5] Chou C, Condron L, Belland JC (2005). A review of the research on Internet addiction.. Educ Psychol Rev..

[6] Velezmoro R, Lacefield K, Roberti JW (2010). Perceived stress, sensation seeking, and college students’ abuse of the Internet.. Comput Hum Behav..

[7] Frangos C, Frangos C, Kiohos A (2010). Internet Addiction among Greek University Students: Demographic Associations with the Phenomenon, using the Greek version of Young’s Internet Addictio.. Int J Econ Sci Appl Res..

[8] Jelenchick LA, Becker T, Moreno MA (2012). Assessing the psychometric properties of the Internet Addiction Test (IAT) in US college students.. Psychiatry Res..

[9] Fu KW, Chan WS, Wong PW, Yip PS (2010). Internet addiction: prevalence, discriminant validity and correlates among adolescents in Hong Kong.. Br J Psychiatry..

[10] Hetzel-Riggin MD, Pritchard JR (2011). Predicting problematic Internet use in men and women: the contributions of psychological distress, coping style, and body esteem.. Cyberpsychol Behav Soc Netw..

[11] Yen JY, Ko CH, Yen CF, Wu HY, Yang MJ (2007). The comorbid psychiatric symptoms of Internet addiction: attention deficit and hyperactivity disorder (ADHD), depression, social phobia, and hostility.. J Adolesc Health..

[12] Cao H, Sun Y, Wan Y, Hao J, Tao F (2011). Problematic Internet use in Chinese adolescents and its relation to psychosomatic symptoms and life satisfaction.. BMC Public Health..

[13] Tonioni F, D'Alessandris L, Lai C, Martinelli D, Corvino S, Vasale M (2012). Internet addiction: hours spent online, behaviors and psychological symptoms.. Gen Hosp Psychiatry..

[14] Fallahi V (2011). Effects of ICT on the youth: A study about the relationship between internet usage and social isolation among Iranian students.. Procedia-Social Behav Sci..

[15] Tsai HF, Cheng SH, Yeh TL, Shih CC, Chen KC, Yang YC (2009). The risk factors of Internet addiction--a survey of university freshmen.. Psychiatry Res..

[16] Ghamari F, Mohammadbeigi A, Mohammadsalehi N, Hashiani AA (2011). Internet addiction and modeling its risk factors in medical students, iran.. Indian J Psychol Med..

[17] Alavi SS, Eslami M, Maracy MR, Najafi M, Jannatifard F, Rezapour H (2010.). Psychometric properties of Young internet addiction test.. J Behav Sci (JBS)..

[18] Widyanto L, McMurran M (2004). The psychometric properties of the internet addiction test.. Cyberpsychol Behav..

[19] Canan F, Ataoglu A, Ozcetin A, Icmeli C (2012). The association between Internet addiction and dissociation among Turkish college students.. Compr Psychiatry..

[20] Lee BW, Stapinski LA (2012). Seeking safety on the internet: relationship between social anxiety and problematic internet use.. J Anxiety Disord..

[21] Ko CH, Hsiao S, Liu GC, Yen JY, Yang MJ, Yen CF (2010). The characteristics of decision making, potential to take risks, and personality of college students with Internet addiction.. Psychiatry Res..

[22] Li Shih-Ming, Chung Teng-Ming (2006). Internet function and Internet addictive behavior.. Comput Hum Behav..

[23] Vizeshfar F (2005). Assessment of the internet addiction between larian net users.. Q J Fund Mental Health..

